# Effectiveness of Exosome Treatment in Androgenetic Alopecia: Outcomes of a Prospective Study

**DOI:** 10.1007/s00266-024-04332-3

**Published:** 2024-08-22

**Authors:** Mert Ersan, Emre Ozer, Ozlem Akin, Pakize Neslihan Tasli, Fikrettin Sahin

**Affiliations:** 1 Plastic, Reconstructive and Aesthetic Surgery Department, Kozyatagi Hospital, Faculty of Medicine, Yeditepe University, Icerenkoy Mahallesi, Hastahane Sokak, 34752 Atasehir, Istanbul, Turkey; 2 Dermatology Department, Kozyatagi Hospital, Faculty of Medicine, Yeditepe University, Icerenkoy Mahallesi, Hastahane Sokak, 34752 Atasehir, Istanbul, Turkey; 3 Genetics and Bioengineering Department, Faculty of Engineering and Architecture, Yeditepe University, Kayisdagi, Inonu Mahallesi, Kayisdagi Caddesi, 34755 Atasehir, Istanbul, Turkey

**Keywords:** Androgenetic alopecia, Exosome, Extracellular vesicles, Hair density, Hair loss, Mesenchymal stromal cells

## Abstract

**Objective:**

Harnessing the regenerative capabilities of stem cell-derived exosomes holds great promise for developing novel hair growth therapies, offering hope for individuals experiencing hair loss or alopecia. This aimed to elucidate the effect of “foreskin-derived mesenchymal stromal cells derived exosome” injection into the scalp on hair density in patients with androgenetic alopecia and the contribution of this treatment on patient satisfaction.

**Method:**

This prospective study included 30 male patients, aged between 22 and 65, with hair type III-VI according to the Norwood-Hamilton scale. Characterization of the stem cell exosomes was performed with the nanoparticle tracking analysis (NTA), hair densities were calculated via digital imaging analysis, and patient satisfaction was questioned with a modified survey.

**Results:**

NTA results showed a characteristic distribution of peaks for exosomes 139.7 ± 2.3 nm in diameter. A statistically significant increase in hair density was observed in the 4th and 12th weeks after treatment (*p *< 0.05). Patient-reported satisfaction revealed a statistically significant difference in the answers given in the 12th week compared to the 4th week (*p *< 0.05). No side effects or complications were observed after exosome injection.

**Conclusion:**

Foreskin-derived mesenchymal stromal cells derived exosome injection increased hair density, with sustained patient satisfaction throughout the study. The exosome application resulted in no side effects.

**Level of Evidence IV:**

This journal requires that authors assign a level of evidence to each article. For a full description of these Evidence-Based Medicine ratings, please refer to the Table of Contents or the online Instructions to Authors www.springer.com/00266.

**Supplementary Information:**

The online version contains supplementary material available at 10.1007/s00266-024-04332-3.

## Introduction

Androgenic alopecia (AGA) occurs through different mechanisms in people with genetic predisposition; its frequency and severity increase with age have different characteristic hair loss patterns in men and women. Since the pathogenesis, clinical features, and treatment management of androgenetic alopecia may differ, evaluating it as male-type and female-type androgenetic alopecia is more accurate. In men, hair in the frontal, vertex, and sometimes temporal areas of the scalp is lost, while the frontal hairline is usually preserved, and hair loss is observed in women’s frontal and vertex areas [1]. The prevalence of androgenetic alopecia is estimated to vary between 23 and 87%. Although the possibility of polygenetic inheritance cannot be excluded, it is thought to show an autosomal dominant transmission with variable penetrance [2].

Androgenic alopecia treatment aims to stop follicular miniaturization and improve hair density. Before starting treatment, patients should be informed about the treatments’ possible effects, side effects, and costs. The patient’s expectations from the treatments should be carefully questioned, and it should be shared with the patient that even if there is no new hair growth, even the cessation of hair loss will be considered a good response to the treatment [3].

Minoxidil is the most used drug in local treatment. Antiandrogens and 5-α reductase inhibitors belong to the class of androgen-dependent treatments. Among the antiandrogens, cyproterone acetate (CPA), spironolactone (SP), and flutamide are used only in female patients due to their potential side effects in male patients. CPA suppresses the production of gonadotropins by inhibiting the release of gonadotropin-releasing hormone. It also blocks androgen receptors. CPA, which is often included in oral contraceptives in combination with androgen and treated with estradiol, is especially preferred in female patients with hyperandrogenism. The basic surgical method is auto transplantation, where hair follicles in the occipital region are transplanted to the frontal, temporal, parietal and vertex regions [1, 4].

Extracellular vesicles are lipid-bound structures secreted by various cell types. They play a crucial role in intercellular communication by transporting various molecules between cells, such as proteins, nucleic acids, and other bioactive molecules. Recent research suggests that these extracellular vesicles, particularly stem cell-derived exosomes, possess remarkable regenerative properties, demonstrating a potential for stimulating hair follicle regeneration and promoting hair growth [5].

Exosomes contain growth factors, cytokines, and microRNAs that can modulate signalling pathways involved in hair follicle development and regeneration. Moreover, stem cell-derived exosomes can interact with target cells by binding to specific membrane receptors, initiating signalling cascades, and promoting hair follicle proliferation and differentiation. Furthermore, the transfer of membrane proteins from exosomes to recipient cells through membrane fusion facilitates the integration of exosomal contents into the cellular machinery of target cells, leading to enhanced hair follicle function and growth [6].

Research into the therapeutic potential of stem cell-derived exosomes for hair regeneration is rapidly advancing, with studies highlighting their involvement in various cellular processes crucial for hair growth. Harnessing the regenerative capabilities of stem cell-derived exosomes holds great promise for developing novel hair growth therapies, offering hope for individuals experiencing hair loss or alopecia [7].

Within the scope of this research, we aimed to elucidate the effect of “foreskin-derived mesenchymal stromal cells derived exosome” injection into the scalp on hair density in patients with androgenetic alopecia and the contribution of this treatment on patient satisfaction.

## Materials and Methods

This prospective study included 30 male patients, aged between 22 and 65, with hair type III–VI according to the Norwood-Hamilton scale. They agreed not to change their hairstyle and would not undergo any hair care or treatment during the study. All procedures followed were under the ethical standards of the responsible committee on human experimentation (institutional and national) and with the Helsinki Declaration of 1975, as revised in 2008. Our study was conducted under strict ethical guidelines and received institutional ethical approval under protocol number 1809. The exosomes used in this study were produced in aseptic conditions under GMP regulations by Yeditepe University Gene and Cell Therapy Excellence Center (YUCTEC), a GMP-compliant laboratory licensed by the Ministry of Health, Turkish Medicines and Medical Devices Agency. All participants provided informed consent. Exosome isolation, preparation, and injection were performed under sterile conditions, and participants were closely monitored for any adverse effects.

Patients using finasteride, dutasteride, steroids, vasodilators, anticonvulsants, beta-receptor blockers, bronchodilators, diuretics, spironolactone, cimetidine, diazoxide, cyclosporine, ketoconazole; patients with a history of surgery for hair loss, such as hair transplantation or scalp reduction; patients with a history of topical steroids or hair growth solutions for hair within the last year; those with uncontrolled blood pressure and blood sugar levels in the last six months, infectious skin diseases or psychiatric disorders, a history of treatment of hyperthyroidism or hypothyroidism, aspartate aminotransferase (AST) or alanine aminotransferase (ALT) serum levels > 80 mg/dL or creatinine (Cr) level > 1.5 mg/dL; and patients who were actively pregnant, breastfeeding, or planning to become pregnant within the next six months were excluded from the study.

### Cell Culture Conditions

Foreskin-derived mesenchymal stem cells (MSCs) were utilized and sourced from the Extracellular Vesicle and Exosome Research Laboratory (EVER Lab) at Yeditepe University. The mesenchymal stem cells used in the article were human foreskin stem cells which were collected from the newborn prepuce tissue in Yeditepe University/Turkey Biotechnology Laboratories as performed by Somuncu et al. [8]. Cells were maintained in Dulbecco’s modified Eagle’s medium *(*DMEM, #41966-029, Invitrogen, Gibco, UK), supplemented with either 10% foetal bovine serum (FBS, #10500-064, Invitrogen, Gibco, UK) and 1% penicillin/streptomycin/amphotericin (PSA, Invitrogen, Gibco, UK) or without FBS. The culture of MSC cells followed the manufacturer’s instructions, utilizing a complete media composed of Basal Medium (#PT-4927) supplemented with the appropriate additives (SingleQuot Kit, #PT-4514). Cells were maintained at 37 °C in a humidified atmosphere containing 5% CO_2_.

### Exosome Isolation

The identification of exosomes was performed in our laboratory, as in the study by Sagrac et al. [9]. The cell culture media is collected from a combination of FBS and antibiotic-free stem cell culture. An ATPS-exosome isolation solution is prepared by blending polyethylene glycol (PEG) and dextran (DEX) at a 7.7:3.3 (w/w) ratio with distilled water. Centrifugation was performed at 10.000 g for 10 min to remove larger contaminants from 20 mL of plant lysate. The supernatant is mixed with the ATPS-exosome isolation solution at a 1:1 volume ratio. Phase separation is induced by centrifuging the mixture at 1.000 g for 10 minutes. Two rounds of removing 80% of the upper PEG-rich phase and substituting it with the upper phase of a washing solution are performed. The washing solution, produced by mixing the exosome isolation solution with distilled water at a 1:1 volume ratio and centrifuging at 1.000 g for 10 min, is utilized. Following the second wash, exosomes are extracted from the phase comprising 10% of the total solution. The collected exosomes are now devoid of cellular debris and can be subjected to subsequent analyses or experiments.

### NTA Analysis

Stem cell exosome concentration measurement and determination of exosome size and density distribution were conducted using Nanosight NS300 (Malvern Panalytical, England) equipped with a 488-nm laser. Exosomes were appropriately diluted to match the recommended concentration range of the instrument, spanning from 20 to 200 points in the frame range. Video recordings were conducted at the 16th camera level with 30-sec intervals between captures. After each capture, the sample was introduced into the flow cell to rinse the previous portion. A total of 10 captures were acquired for each sample—subsequent analysis involved employing suitable threshold settings. The video capture and analysis processes were executed utilizing NTA software version 3.4.

### Surface Antigen Measurement

Exosomal surface antigens were assessed using flow cytometry. Initially, approximately 10^9^ nanoparticles (~5 μg) of exosomes were bound to 5 μL of aldehyde/sulphate latex beads (ThermoFisher, A37304), each with a concentration of 4% w/v and a size of 4 μm. This mixture was then incubated for 15 min on a shaker at room temperature. After the incubation period, Exo-Bead suspension was divided into four tubes for antibody incubation. Conjugated antibodies targeting common exosome markers, including CD9 (Biolegend, 124808), CD63 (Biolegend, 143904), and CD81 (Biolegend, 349506, USA), were added to each sample at a dilution of 1:1000 and allowed to incubate overnight. Finally, exosome analysis was performed via flow cytometry using a Becton Dickinson (BD) FACSCalibur Flow Cytometry System (Becton Dickinson, San Jose, CA, USA).

### Evaluation of AGA

Before the exosome injections of 30 male patients with androgenetic alopecia, frontal and vertex regions where hair loss occurred on the scalp were imaged with a digital camera (Canon Eos 5D Mark II, Canon Inc. Tokyo, Japan). To ensure measurements from the same point during controls, some topographic points were determined. For 2 points in the frontal region, the points within 5 cm of the hairline at the left and right mid-pupil lines were considered, and for the one point at the vertex, 2 cm medial to the hair whorl was taken as the basis. An area of 1 cm^2^ from each of the mentioned areas was selected, and × 40 magnification images of those areas were taken with digital dermatoscopy (MoleMax HD, Derma Medical Systems, Austria). These dermatoscopic images recorded hair densities (hair count/cm^2^) with Trichoscan (TrichoLab GmbH, Germany). The average values of 3 regions (2 frontal and 1 vertex points) were taken. After the imaging, the application was performed: A total of 3 mL of exosomes (2 mL to the frontal and 1 mL to the vertex region) was injected using the napage technique (10^10^ extracellular vesicles in 1 mL). Patients were observed for possible side effects for 1 h after the procedure. Patients were instructed not to wash their hair and avoid heavy activities for 1 day after the injection. The patients were called for control at the 4th and 12th weeks after the injection. During these sessions, photographs of the same areas were taken with the same digital camera, from the same distance and under the same light and flash (1/200 s; f/6, 3; ISO 160). During the controls, the same topographic points were found (2 frontal and 1 vertex point), and these areas were imaged with digital dermatoscopy under × 40 magnification. Hair densities were recorded using Trichoscan analyses, and the averages of the 3 treated areas were taken.

Additionally, at the 4- and 12-week check-ups, a hair growth survey, as defined by Barber et al. [10] , was modified and administered to the patients. In this modified survey, patients were asked two questions. Question one was, “Has your hair loss decreased?” while the second question was, “Have you noticed new hair growing?” Participants answered both questions: “1-I strongly agree, 2-I agree, 3-I am undecided, 4-I disagree, 5-I strongly disagree”.

### Statistical Analysis

All statistical analyses were performed using SPSS (Statistical Package for Social Sciences) version 29.0 software (IBM SPSS Inc., Armonk, NY, USA). Descriptive statistics were presented to measure the clinical characteristics of the results. These were mean, median, standard deviation (SD), minimum, and maximum for continuous variables, and frequency and percentage for categorical variables. A two-tailed Kolmogorov–Smirnov test was applied to examine whether the continuous quantitative variables follow a Gaussian distribution. The Wilcoxon signed rank test or paired sample *T* test was used to compare two dependent groups. The “*p*” value of < 0.05 was accepted for statistical significance.

## Results

A total of 30 male adults with alopecia areata were enrolled in this prospective research. The average age of the participants was 34.65 years (range 22–65 years). All patients completed the study and the follow-up period with no dropouts. No side effects or complications were observed immediately after exosome injection or during follow-up.

### Exosome Identification

**Fig. 1:**
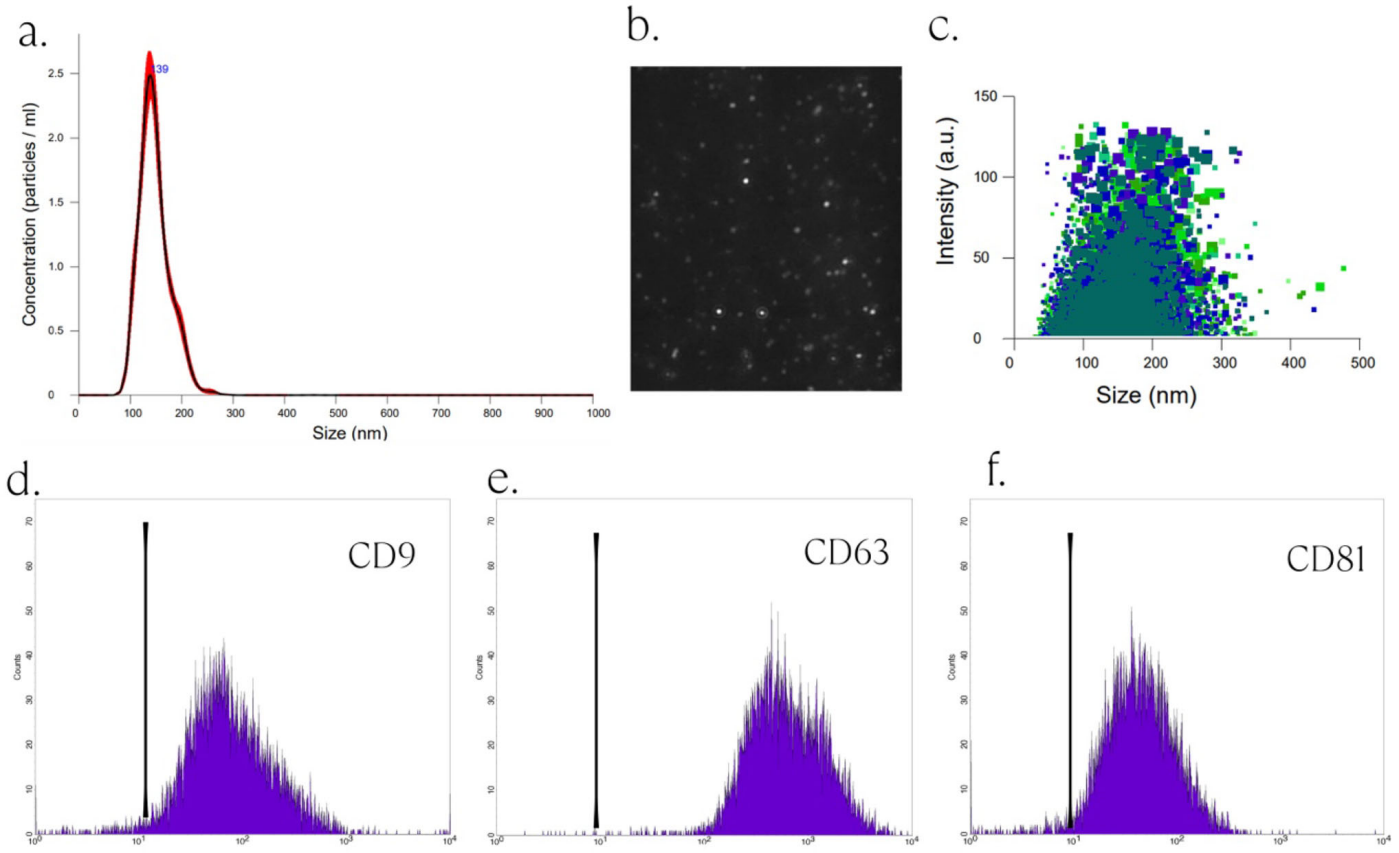
Characterization of stem cell exosome. **a** Size and concentration measurements of stem cell exosome with NS300 NTA system with 15 different reads for 30 sec. each reads. **b** Brownian motion image of C-Exo particles. **c** Intensity and size distribution. **d** Surface antigen CD9. **e** Surface antigen CD63. **f** Surface antigen CD81

NTA results showed a characteristic distribution of peaks for exosomes 139.7 ± 2.3 nm in diameter (Fig. 1a). Exosome sizes were measured between 30 and 200 nm (Fig. 1a–c). Exosomes have a homogeneous structure with a round-shaped morphology (Fig. 1b, c). Brownian motion of exosomes can be seen in Video 1. The isolated stem cell exosome particle number was 1.59 10^10 ^± 10^8^ nanoparticle/mL. The surface antigens CD9, CD63, and CD81, assessed using flow cytometry, are shown (Fig. 1d–f), respectively.

### Digital Image Analysis and Hair Density Improvement

**Fig. 2:**
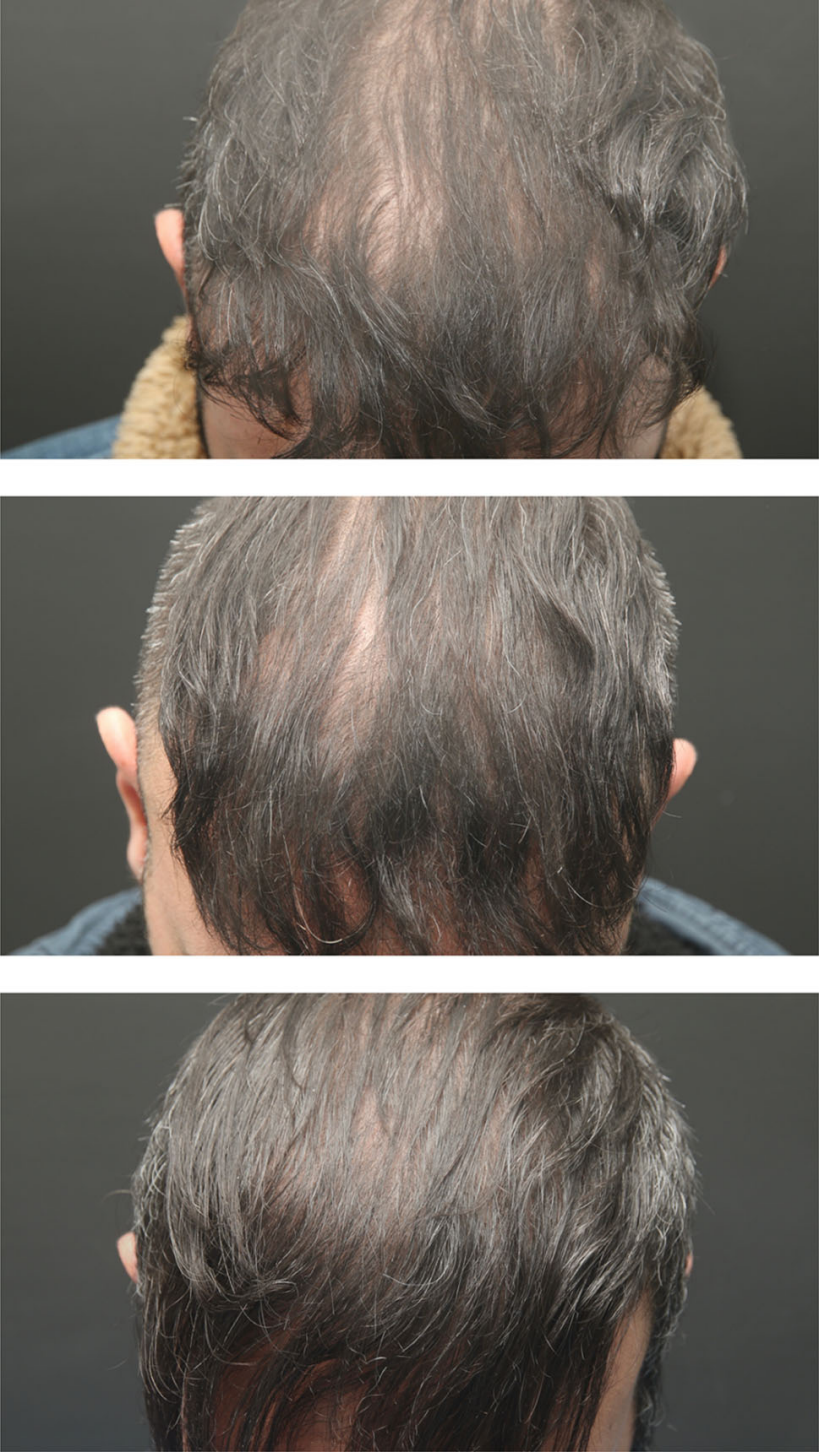
The frontal area of the patient who received a single dose of exosome injection, respectively (from top to bottom), pre-treatment, four weeks after treatment, and 12 weeks after treatment. The average hair density (hair count/cm^2^) from two designated frontal points was 128, 155, and 162, respectively

**Fig. 3:**
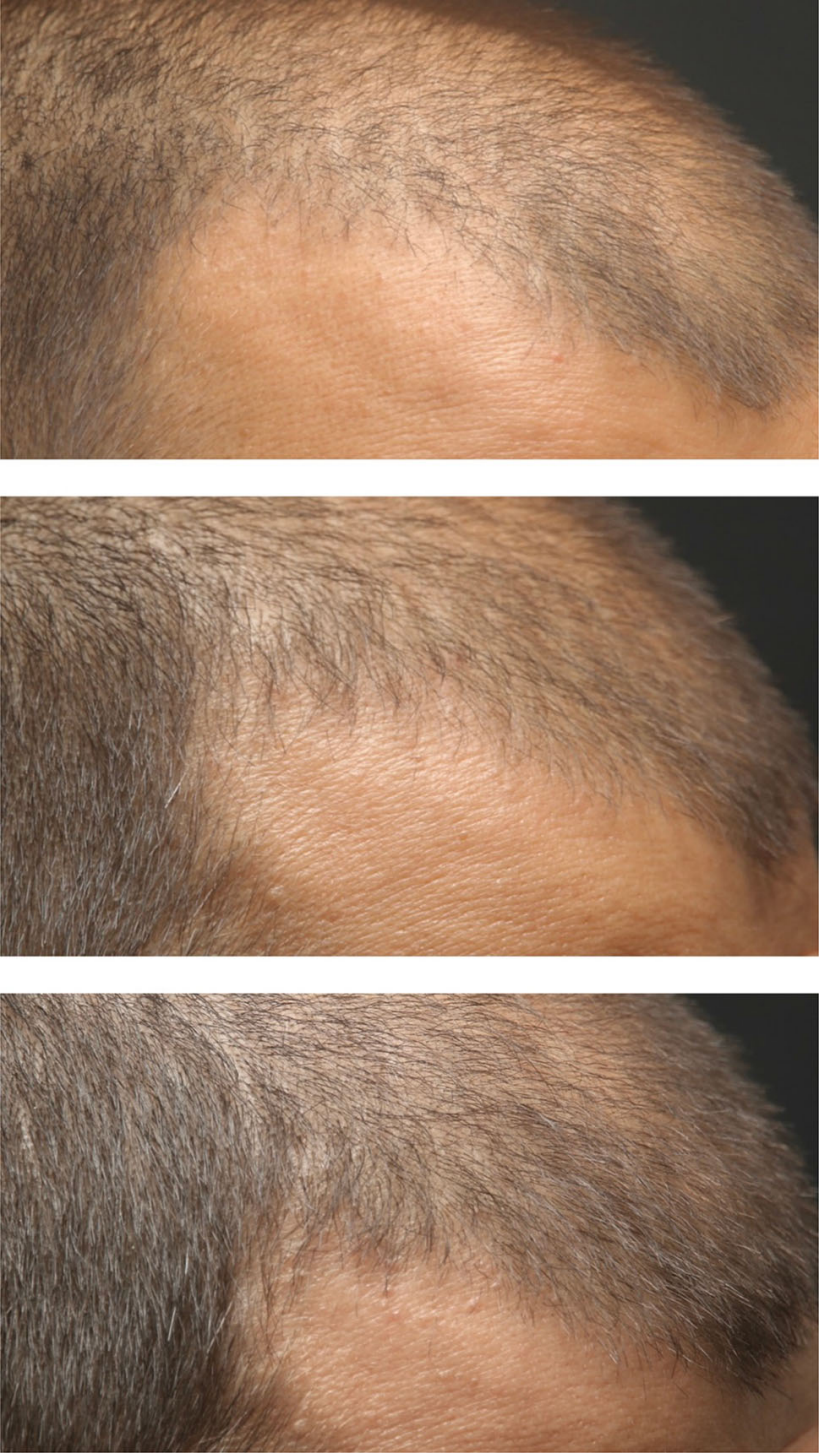
A close-up view of the right frontal area of the patient taken from the right oblique angle, who received a single dose of exosome injection, respectively (from top to bottom) pre-treatment, 4 weeks after treatment, and 12 weeks after treatment. The hair density (hair count/cm^2^) taken from a designated frontal point on the right side was 151, 164, and 170, respectively

**Fig. 4:**
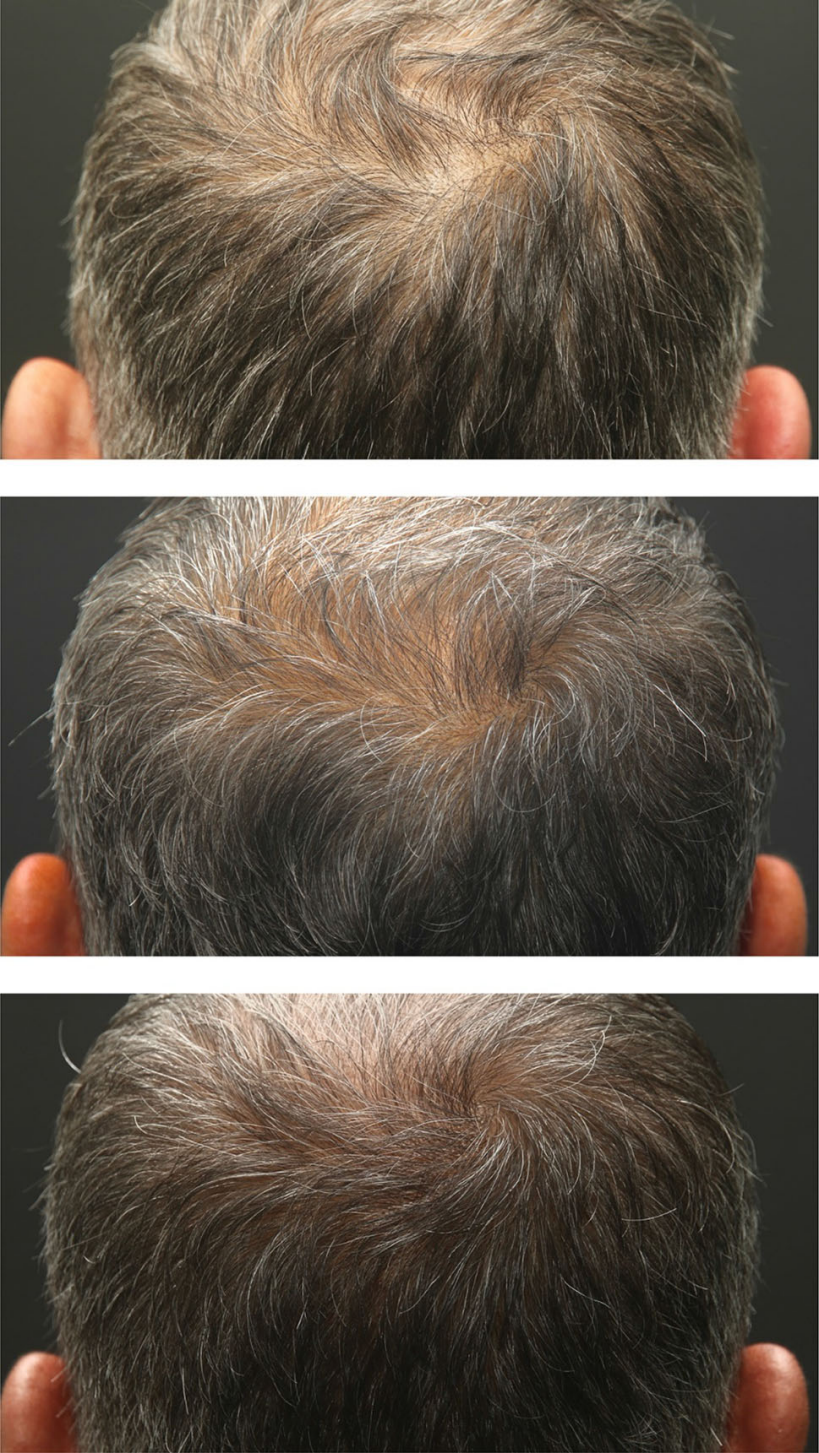
The vertex area of the patient who received a single dose of exosome injection, respectively (from top to bottom) pre-treatment, four weeks after treatment, and 12 weeks after treatment. The average hair density (hair count/cm^2^) taken from designated vertex points was 121, 128, and 133, respectively

**Fig. 5:**
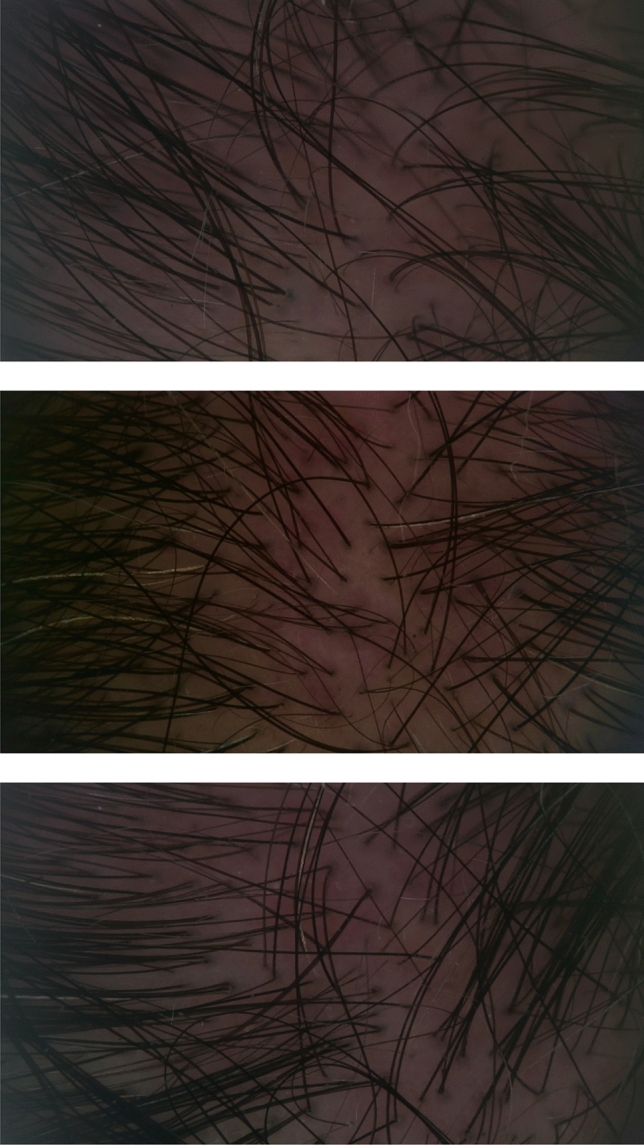
The dermatoscopic image of the left frontal area of the patient (different patient from those in the macroscopic images) who received a single dose of exosome injection, respectively (from top to bottom) pre-treatment, four weeks after treatment and 12 weeks after treatment. The hair density (hair count/cm^2^) taken from a designated vertex point was 139, 172, and 175, respectively

Two independent observers evaluated the macroscopic photographs. We observed significant improvements in both the macroscopic view (Figs. 2, 3, 4) and the dermatoscopic images (Fig. 5)*.*

According to the patients’ digital dermatoscopy images and Trichoscan analyses, the distribution of hair density at the 4th week after treatment compared to before treatment, at the 12th week after treatment compared to before treatment, and the 4th and 12th weeks after treatment are denoted in Table 1. A statistically significant increase was observed in the 4th and 12th weeks after treatment compared to pre-treatment (*p* < 0.05). The mean hair density increased from 149.7 ± 13.7 hairs/cm^2^ at pre-treatment to 153.6 ± 16.8 hairs/cm^2^ at the 4th week (*p* = 0.043) and further to 157 ± 18.3 hairs/cm^2^ at the 12th week (*p* = 0.002).

**Table 1:** Distribution of hair density (hair count/cm^2^) results of the patients before treatment, 4th week and 12th week after treatment

Hair density (hair count/cm^2^)	Mean ± SD^*+*^	Median (min–max)	*p* value
Pre-treatment	149.7±13.7	149.5 (121-187)	*0.043
4. week	153.6±16.8	154.5 (118-194)	
Pre-treatment	149.7±13.7	149.5 (121-187)	**0.002
12. week	157±18.3	156 (119-202)	
4. week	153.6±16.8	154.5 (118-194)	**0.002
12. week	157±18.3	156 (119-202)	

* *p* < 0.05; ** *p* < 0.01

^*+*^*SD* standard deviation

### Survey Results and Patient Satisfaction

The distribution of the answers to the question “Has your hair loss decreased?” given in the 12th week according to the answers given in the 4th week is elaborated in Table 2. There was a statistically significant difference in the answers given in the 12th week compared to the 4th week (*p* < 0.05). According to the answers given by the patients in the 4th week, a positive change was observed in the 12th week. The distribution of the answers to the question “Have you noticed new hair growing?” given in the 12th week according to the answers given in the 4th week is elaborated in Table 3. A statistically significant difference was seen in the answers given in the 12th week compared to the 4th week (*p* < 0.05). According to the patients’ answers in the 4th week, a positive change was observed in all but five patients in the 12th week.

**Table 2:** The distribution of patients’ responses regarding the “decrease in hair loss” at the 4th and 12th weeks after treatment

	Has your hair loss decreased in the 12th week?					*p* value
	I agree	I don’t agree	Undecided	Disagree	Strongly disagree	
Has your hair loss decreased in the 4th week? *n* (%)						***<0.001
I agree	2 (50)	2 (50)	0 (0)	0 (0)	0 (0)	
I’m undecided	3 (27.3)	4 (36.4)	3 (27.3)	0 (0)	1 (9.1)	
I disagree	4 (44.4)	3 (33.3)	2 (22.2)	0 (0)	0 (0)	
I strongly disagree	3 (50)	2 (33.3)	0 (0)	1 (16.7)	0 (0)	

*** *p* < 0.001

**Table 3:** The distribution of patients’ responses regarding the “new hair growth” at the 4th and 12th weeks after treatment

	Have you noticed new hair growing in the 12th week? *n* (%)				*p* value
	Strongly agree	Agree	Undecided	Disagree	
Have you noticed new hair growing in the 4th week? *n* (%)					*0.035
Strongly agree	11 (73.3)	4 (26.7)	0 (0)	0 (0)	
Agree	2 (14.3)	8 (57.1)	4 (28.6)	0 (0)	
Undecided	0 (0)	0 (0)	0 (0)	1 (100)	

* *p* < 0.05

## Discussion

Exosomes can be obtained from all body fluids and play an important role in many biological functions, such as intercellular communication, signal transmission, genetic material transfer, and regulation of the immune response. Understanding that exosomes have such functions has revealed different areas of nanovesicle use. The first of these is diagnosis due to their content and role in the pathogenesis of different diseases [11]. Another one is due to the immune regulatory properties of exosomes, which even lead to cancer treatment. In addition to all these roles, exosomes play important roles in immune responses and ensure immune homeostasis. Studies have shown that exosomes of different cell origins have suppressive and activating effects on immune cells, which can have multiple effects (pleiotropic). The pleiotropic effects of exosomes, depending on the state of the cells from which they originate, enable the emergence of different biomedical applications of these natural nanovesicles [12].

MSC exosomes are also promising in hair restoration as they contain potent cytokines and growth factors that promote hair growth. Early studies have shown that MK exosomes induce the proliferation/migration of human dermal papilla cells and secretion of VEGF and IGF-1 in vitro [13]. Rajendran et al. (2017) injected dermal papilla cell-derived exosomes in mice. They showed that it accelerated the onset of the anagen phase of the hair follicle, delayed the catagen phase, and stimulated the expression of beta-catenin and “sonic-hedgehog” growth factors [14]. Kwack et al. [15] reported that exosome treatment increased average hair density and thickness. The study stated that exosomes stimulated hair follicle proliferation, accelerated the transition from the telogen to the anagen phase, and protected hair follicle cells against reactive oxygen species. The authors injected exosomes into the scalp, similar to platelet-rich plasma (PRP), and treatments can be adjusted to the degree of hair loss.

The spheroid form of exosomes derived from dermal papilla triggered the transition from the telogen phase to the anagen phase better compared to minoxidil treatment [16]. Dermal papilla-derived exosomes provide many benefits to overall hair growth. The presence of “miRNA” ensures the proliferation/differentiation of stem cells and the formation of longer hair shafts. Other source cells studied for hair growth include mesenchymal stem cells and keratinocytes. Yang et al. [17] stated that the microneedle-based transdermal drug delivery approach showed increased effectiveness compared to subcutaneous exosome injections and topical UK5099 application. Hair follicle-derived MSCs have also been shown to reduce inflammation and decrease hair loss in vitro in mice with AA, an autoimmune type of hair loss [18].

Festa et al. [19] indicated that the number of adipocyte precursor cells changed with the hair cycle. The cell number peaked in the skin during follicular stem cell activation (anagen) and decreased during the catagen phase. They also reported that mature adipocytes and preadipocytes have been defined as skin niche cells that regulate hair follicle stem cell activity. ASCs facilitate the production and secretion of growth factors such as vascular endothelial growth factor (VEGF), transforming growth factor (TGF-*β*), hepatocyte growth factor (HGF), platelet-derived growth factor (PDGF), placental growth factor (PlGF), and basic fibroblast growth factor (bFGF) [20]. Intradermal injections of adipose-derived stem cell conditioned media (ADSC-CM) have also been investigated previously. A retrospective cohort of 27 individuals with female-pattern hair loss (FPHL) treated with a single ADSC-CM intradermal injection showed that ADSC-CM application promoted hair density and thickness in these patients without adverse reactions [21].

Clinical studies on MSC-derived exosomes have shown promising safety profiles, with no significant adverse effects, such as anaphylaxis, reported in several trials. For example, MSC-derived exosomes in various clinical settings have demonstrated safety without inducing significant adverse effects, including immune reactions or anaphylaxis [22–24]. These findings support the safety of exosome therapy, although continuous monitoring and preparedness for managing potential side effects are essential. Although no side effects or complications were observed immediately after the exosome injection or during follow-up, we took several precautionary measures to ensure patient safety. Before the procedure, we carefully checked each patient for potential allergies or risk factors related to exosomes. This included a thorough medical history, focusing on any known allergies, previous allergic reactions to medications or treatments, and family history of allergies. Patients were observed for 1 hour post-procedure to monitor for any immediate adverse reactions, including anaphylaxis. Our clinic has emergency medical supplies and medications to manage anaphylaxis, such as epinephrine auto-injectors, antihistamines, corticosteroids, and oxygen. Our staff members are trained to recognize and respond to anaphylactic reactions promptly. In the event of an anaphylactic reaction, our protocol includes immediately administering intramuscular epinephrine, providing supplemental oxygen, administering antihistamines and corticosteroids, and arranging for emergency medical transport if necessary.

Stevens et al. [25] evaluated the efficacy of stromal vascular fraction (SVF) injections in combination with platelet-rich plasma (PRP) in the upper scalp of AGA patients. In their study, hair density significantly increased after 6 weeks from an average baseline value of 157–177 hairs/cm^2^ (*p* = 0.013). At 12 weeks, the hair density increased to 185 hairs/cm^2^ (*p* < 0.001). Regarding their research outcomes, they admitted that a single treatment of platelet-rich stroma injected in the scalp of patients with AGA significantly increased hair density within 6–12 weeks [25]. Tak et al. [20] conducted a randomized controlled trial on 38 AGA patients (29/38 males) who self-applied the ADSC constituent extract topical solution twice daily over the scalp with fingers and analyzed hair count and thickness at 16 weeks. In the study, the hair density, 139.7 ± 2.3 hairs/cm^2^, increased to 153.6 ± 16.8 hairs/cm^2^ at week 16 (*p* < 0.05). They declared that applying adipose-derived stem cell constituent extracts topical solution enhanced hair regrowth by increasing hair density and thickness while maintaining adequate treatment safety [20]. Our study observed a statistically significant increase in hair density at the 4th and 12th weeks post-treatment with exosome injections in digital dermatoscopy images and Trichoscan analyses. The mean hair density increased from 149.7 ± 13.7 hairs/cm^2^ at pre-treatment to 153.6 ± 16.8 hairs/cm^2^ at the 4th week (*p* = 0.043) and further to 157 ± 18.3 hairs/cm^2^ at the 12th week (*p* = 0.002). Comparing these results, it is evident that the exosome injections in our study demonstrated comparable efficacy to the PRP and SVF injections used in the study by Stevens et al. (2018) and the adipose-derived stem cell constituent extract topical solution used by Tak et al. [20, 25]. These studies reported significant increases in hair density over their respective timeframes, aligning closely with our findings.

As mentioned above, these comparisons highlight the potential of exosome therapy as a viable alternative for hair restoration in patients with androgenetic alopecia. The consistent efficacy across different treatment modalities suggests that regenerative therapies, including exosome injections, hold significant promise for clinical application. Further research with larger sample sizes and control groups would be beneficial to confirm these findings and optimize treatment protocols.

Apart from previous literature, patient satisfaction could be attributed as the strength of this research. Patients reported decreased hair loss and improved growth in the 4th and 12th weeks after exosome injection.

We believe our study has some limitations. The first is the absence of a control group. This study could pave the way for a larger patient population study, including a control group created with saline injections. Another limitation of the study is that it does not evaluate the effects of repeated exosome injections. We believe our study will shed light on further research investigating the effects of repeated exosome injections at certain intervals.

## Conclusion

Foreskin-derived mesenchymal stromal cells derived exosome injection increased hair density in the first and third months after application. The patient satisfaction was sustained throughout the study. Last but not least, the exosome application resulted in no side effects or withdrawals. There is a need for larger population studies where exosome injections are compared with different hair loss treatment methods, and the efficacy of repeated exosome injections is also evaluated.

## Supplementary Information

Below is the link to the electronic supplementary material.

Supplementary file1 (MP4 990 KB)
